# Defective alterations in the collagen network to prostacyclin in COPD lung fibroblasts

**DOI:** 10.1186/1465-9921-14-21

**Published:** 2013-02-14

**Authors:** Anna-Karin Larsson-Callerfelt, Oskar Hallgren, Annika Andersson-Sjöland, Lena Thiman, Johan Björklund, Josefine Kron, Kristian Nihlberg, Leif Bjermer, Claes-Göran Löfdahl, Gunilla Westergren-Thorsson

**Affiliations:** 1 Unit of Lung Biology, Department of Experimental Medical Sciences, BMC D12, Lund University, 221 84, Lund, Sweden; 2 Department of Respiratory Medicine and Allergology, Skåne University Hospital, Lund University, Lund, Sweden

**Keywords:** Chronic obstructive pulmonary disease, Collagen I, Fibroblast, Prostacyclin, Proteoglycans, Decorin, Biglycan, Proliferation, Fibroblast gel contraction, Transforming growth factor β

## Abstract

**Background:**

Prostacyclin analogs are potent vasodilators and possess anti-inflammatory properties. However, the effect of prostacyclin on extracellular matrix (ECM) in COPD is not well known. Collagen fibrils and proteoglycans are essential ECM components in the lung and fibroblasts are key players in regulating the homeostasis of ECM proteins. The aim was to study the synthesis of prostacyclin and its effect on fibroblast activity and ECM production, and in particular collagen I and the collagen-associated proteoglycans biglycan and decorin.

**Methods:**

Parenchymal lung fibroblasts were isolated from lungs from COPD patients (GOLD stage IV) and from lungs and transbronchial biopsies from control subjects. The prostacyclin analog iloprost was used to study the effect of prostacyclin on ECM protein synthesis, migration, proliferation and contractile capacity of fibroblasts.

**Results:**

TGF-β_1_ stimulation significantly increased prostacyclin synthesis in fibroblasts from COPD patients (p < 0.01), but showed no effect on fibroblasts from control subjects. Collagen I synthesis was decreased by iloprost in both control and COPD fibroblasts (p < 0.05). Conversely, iloprost significantly altered biglycan and decorin synthesis in control fibroblasts, but iloprost displayed no effect on these proteoglycans in COPD fibroblasts. Proliferation rate was reduced (p < 0.05) and contractile capacity was increased in COPD fibroblasts (p < 0.05) compared to control fibroblasts. Iloprost decreased proliferative rate in control fibroblasts (p < 0.05), whereas iloprost attenuated contraction capacity in both COPD (p < 0.01) and control fibroblasts (p < 0.05).

**Conclusions:**

Iloprost reduced collagen I synthesis and fibroblast contractility but did not affect the collagen-associated proteoglycans or proliferation rate in fibroblasts from COPD patients. Enhanced prostacyclin production could lead to improper collagen network fibrillogenesis and a more emphysematous lung structure in severe COPD patients.

## Background

COPD is a chronic obstructive lung disorder with emphysematous lesions in the distal lung. The pathophysiology of COPD is complex and involves airway inflammation and structural changes of the pulmonary system [1]. Alterations in vascular and parenchymal structures may impair gas exchange in alveoli [2] and involve systemic complications, as pulmonary hypertension, that is associated with increased disease severity and mortality [3]. Prostacyclin is a key mediator in regulating vascular tone and prostacyclin analogs such as iloprost, are frequently prescribed to decrease pulmonary arterial pressure, as they are potent vasodilators and possess anti-inflammatory properties [4]. Iloprost treatment has been shown to have favorable acute effects on gas exchange and lung function in a small subset of COPD patients [5,6]. Prostacyclin is formed from arachidonic acid by the cyclooxygenase (COX) pathway and prostaglandin I synthase [7] and binds to prostacyclin (IP) receptors, which activate adenylyl cyclase and generating increased cAMP levels in target cells [8]. Prostacyclin has been implicated to play a major role in tissue repair and remodeling processes by inhibiting profibrotic responses of fibroblasts [9,10]. However, contrasting processes have been described in COPD, such as a degradation of the extra cellular matrix (ECM) in parenchymal compartments, but enhanced deposition of ECM in bronchioles [11]. Collagen fibrils and the specific collagen-associated proteoglycans decorin and biglycan are essential ECM proteins, having both organizational and structural roles in the network of the pulmonary architecture [12]. Collagen I is the predominant fibrillar collagen in the lung and binds to decorin [13]. Fibroblasts are the main source of alveolar connective tissue and are key players in regulating the homeostasis of ECM [12] by constituting a rich source of growth factors and inflammatory mediators, including prostacyclin [14]. Abnormal fibroblast activation may therefore cause pathological tissue remodeling [12]. However, little is known regarding the synthesis and the effect of prostacyclin on the ECM and collagen network organization in COPD. A decreased production of prostacyclin has been correlated to increased fibroblast activity in lung fibrosis [15,16], implicating that prostacyclin could be involved in on-going remodeling processes in COPD. The aim was therefore to study the synthesis of prostacyclin and its effect on ECM production and collagen network in pulmonary fibroblasts. Other markers of fibroblast activity, such as proliferation rate, contractility and migration capacity were also investigated. Primary distal lung fibroblasts obtained from patients with COPD and control subjects were used in this study. Changes in ECM were assessed by the production of collagen I and the collagen-associated proteoglycans decorin and biglycan after stimulations with the prostacyclin analog iloprost. Transforming growth factor (TGF)-β_1_, known as a general inducer of remodeling [12], was added to stimulate fibroblast activity and mimic remodeling processes.

## Methods

### Study subjects

Patients (n = 7) suffering from very severe COPD (spirometric GOLD stage IV) who were undergoing lung transplantation at Lund University Hospital 2006-2008 were included in this study [17]. The patients had quit smoking at least 6 months before the lung transplantation. All patients were given glucocorticoids on a regular basis and in combination with other medicines. Control subjects were recruited from two different sources. First, lung explants from healthy donors (n = 4) with no history of lung disease [18] could be included in this study as no matched recipients were available. Second, non-smokers (n = 5) with no history of smoking or other lung diseases were included in the study [19] (Table 1)*.* Written consent was obtained from all participants or from the closest relative in the present study. The study was approved by the Swedish Research Ethical Committee in Lund (FEK 213/2005, FEK 91/2006 and FEK 413/2008).

**Table 1:** Control subjects and COPD patients in the study

**Characteristics**	**Controls**		**COPD**	
No.	5 + 4^*^		7	
Age (range)	28^*^	(24–34)	62	(53–66)
Pack years (range)	0^*^		40	(25–60)
Gender, M/F in%	33/67		29/71	
**Lung function**				
FEV_1_	4.2^*^	(3.3–5.4)	0.53	(0.4–0.8)
FEV_1_% predicted	102.2^*^	(84–116)	19.3	(14–24)
FVC	5.1^*^	(4.2–6.2)	1.8	(1.3–2.5)
FEV_1_/FVC ratio	82^*^	(71–93)	29	(20-35)
DLCO	-		1.4	(1.4–1.5)^§^
DLCO% predicted	-		27	(21–42)^§^

^*^Values are only from five of the control subjects. The other four subjects were lung donors with no former history of lung disease. Of the lung donors, three out of four had the age interval 46-65 years old and one between 31-45 years old and one of them was an ex-smoker with 7.5 pack years. There were no other lung function parameters available for these individuals. ^§^Values from three patients. Data is presented as mean (range).

### Lung explants and primary distal lung fibroblast cultures

Parenchymal (distal) lung fibroblast cultures were isolated from lung explants from COPD patients after lung transplantation as previously described [17]. Control lung fibroblasts were isolated from lung explants from donors after lung transplantation [18] and from transbronchial biopsy samples from control subjects as previously described [19]. Importantly, alveolar parenchymal specimens from lung explants were collected 2-3 cm from the pleura in the lower lobes, equivalent to the location where the transbronchial biopsies were obtained. Vessels and small airways were removed from the peripheral lung tissues. From biopsy and explant samples of similar size, cultures of primary fibroblast-like cells were established. Briefly, transbronchial biopsies and parenchymal specimens were transferred to cell culture medium immediately after sampling. Parenchymal pieces from biopsies and parenchymal specimens were cut into small pieces that were allowed to adhere to the plastic of cell culture flasks for 4 h and were then kept in cell culture medium in 37°C cell incubators until there were outgrowths of cells with morphology typical for fibroblasts, i.e. cells had a spindle-like shape and several protrusions. Primary fibroblasts were cultured in Dulbecco’s Modified Eagle’s Medium (DMEM) supplemented with 10% fetal Clone III serum (Hyclone, Logan, UT, US), 1% L-glutamine, 0.5% gentamicin and 5 ug/ml amphotericin B (all from Gibco BRL, Paisley, UK). The fibroblast cultures were stained with specific antibodies to verify the mesenchymal identity and to estimate the purity, as previously described [17-19]. Isolated primary fibroblasts were split 1:2 at expansions and were used in passages 4–7 for further experiments.

### Immunocytochemistry and immunohistochemistry

Fibroblasts (7000 cells/well) grown overnight on chamber slides were fixed in 4% paraformaldehyde. The lung tissue explants were fixed in 4% buffered formalin, embedded in paraffin and sectioned in 5 μm thick sections for further analysis. The lung tissue sections were pretreated by heat induced antigen retrieval and non-specific binding was blocked with 5% BSA in TBS. The fixated cells and the lung sections were incubated with a mouse anti-human monoclonal antibody to IP receptor (ab60706, Abcam, Cambridge, MA, US) over night at 4°C and further incubated with a corresponding secondary antibody conjugated with Alexa fluorocrome 488 (Molecular Probes, Eugene, OR, US) in TBS solution containing 1% goat serum (Vector Laboratories, Burlingame CA, US). The nuclei were visualized with DAPI (Invitrogen Corp, Carlsbad, CA, US). Image analyses were performed with Nikon Eclipse microscope, camera Nikon DS-Qi1Mc and software program NIS-Elements AR 3.0 (Nikon, Tokyo, Japan).

### Analysis of prostacyclin and TGF-β_1_ synthesis

Prostacyclin synthesis was measured in the fibroblast medium as the stable prostacyclin metabolite 6-keto PGF_1α_ by a commercially available enzyme immune assay kit (Cayman Chemicals, Limhamn, Sweden). Detection limit for 6-keto PGF_1α_ was 6 pg/mL. TGF-β_1_ production was measured in the cell culture medium by a commercially available ELISA kit for activated human TGF-β_1_ (R&D Systems, Abingdon, England). Detection limit for TGF-β_1_ was 4.6 pg/mL.

### Analysis of collagen I and proteoglycan synthesis

Collagen I synthesis was analyzed in the cell culture medium by measuring the newly synthesis of the N-terminal propeptide of type I procollagen (PINP) by a commercial radio immune assay kit (Orion Diagnostica, Espoo, Finland). Briefly, lung fibroblasts were cultured in 6-well plates. Cells were pretreated with the COX inhibitor indomethacin (3 μM) (to avoid interactions with endogenously produced prostanoids) for 10 min and then stimulated with iloprost (1000 nM) (Cayman Chemicals, Limhamn, Sweden) in combination with TGF-β_1_ (10 ng/ml) (R&D Systems, Abingdon, England) for 24 hours in duplicates. Detection limit was 2 μg/L. Proteoglycan production in fibroblasts was determined as previously described [20,21]. Briefly, lung fibroblasts were cultured in 6-well plates. Cells were incubated in low sulphate DMEM (Gibco BRL, Paisley, UK), pretreated with the COX inhibitor indomethacin (3 μM) for 10 min and then stimulated with different concentrations of iloprost (10, 100 or 1000 nM) (Cayman Chemicals, Limhamn, Sweden) in combination with TGF-β_1_ (10 ng/ml) (R&D Systems, Abingdon, England) in sulfate^35^-containing DMEM for 24 hours in duplicates. Cell medium with 0.4% serum was used as a control of basal activity. Proteoglycan synthesis was quantified by [^35^S]-sulfate incorporation into glycosoaminoglycan side-chains measured on a scintillation counter (Wallac; Perkin Ellmer, Boston, MA, US). Individual proteoglycans were separated by ion exchanger DEAE52 and SDS-PAGE and then quantified using densitometry. The various proteoglycans have previously been identified by mass spectrometry [22]. Proteoglycan production in the medium was related to the total amount of protein in the corresponding cell layer. The amount of proteins in the cell lysate was analyzed by a commercially available protein assay, which constitutes a colorimetric assay with BSA as a standard reference for measuring total protein concentrations (Bio-Rad Laboratories, Hercules, CA, US). The concentration of 10 ng/ml TGF-β was chosen since this concentration has previously been shown to induce a stable and long lasting production of proteoglycans in lung fibroblasts [23].

### Cell proliferation

Cell proliferation rate was determined as previously described [24]. Cells were plated in 96-well plates (Cellstar, Monroe, NC) for 6 hours and then stimulated (5 wells/stimulation) with medium containing 0.4% serum, indomethacin (3 μM) and iloprost (100 nM) for 24 and 48 hours. 10% serum was used as a positive control. Cells were fixated in 1% glutaraldehyde (Sigma-Aldrich, St. Louis, MO, US) stained with 0.1% crystal violet (Sigma-Aldrich, St. Louis, MO, US) and incubated over night with 1% Triton X (Merck, Darmstadt, Germany). Changes in proliferation rate were quantified with a spectrophotometer plate reader at absorbance 595 nm. This method has been shown to be equivalent to cell counting with a Coulter counter [25]. All proliferation experiments are the mean of quintuplicate.

### Migration assay

Migration of cultured fibroblasts was analyzed as previously described [26]. Fibroblasts (30,000 cells) were cultured within a cloning cylinder for 6 hours in medium with 0.4% serum. The cylinder was removed and the cells were stimulated with medium containing 0.4% serum, indomethacin (3 μM) and iloprost (100 nM). 10% serum was used as a positive control. The fibroblasts were allowed to migrate for 24 h. The cells were fixed with 1% glutaraldehyde and then stained with 0.5% crystal violet for 2 hours. Migration capacity was then measured and analyzed with Nikon Eclipse microscope, camera Nikon DXm1200C and software program NIS-Elements AR 3.0 (Nikon, Tokyo, Japan). All migration assays are the mean of triplicates.

### Fibroblast gel contraction assay

The gels were prepared as previously described [18] using a modified form of a previous protocol [27]. Briefly, 96-well cell culture plates (Cellstar, Monroe, NC) were coated with 1% BSA overnight and were then washed with PBS. Fibroblasts, suspended in DMEM (1,000,000 cells/ml), were added to collagen type I solution (PureCol, Inamed Biomaterials, Fremont, CA) in the relation 1:9 (v/v). The final cell density was 100,000 cells/ml. Indomethacin (3 μM) and iloprost (1000 nM) were added to cell suspensions immediately before they were mixed with the collagen solution. 100 μl cells in collagen solution were added to each well and the collagen gels were polymerized for 1 hour at 37°C. After polymerization, 100 μl of DMEM supplemented with 0.4% serum and 1% glutamine was gently added to each well. Gels were released with a spatula 4 hours after polymerization and were then photographed with a camera (Sony, Tokyo, Japan). The gel area at this point was used as the initial area. The gel area was then monitored over time and compared to the initial area after 48 hours. All gel contraction experiments are the mean of triplicates.

### Data analysis and statistical procedures

Data are shown for individual subjects in absolute values and presented as median. The non-parametric Mann Whitney test or Wilcoxon signed rank test was used to compare statistical differences between two groups. One or two way repeated measurement ANOVA on ranks followed by the non-parametric post hoc test Dunn was used to compare differences between more than two groups. Differences were considered to be statistically significant at p < 0.05. All analyses were performed using GraphPad Prism 5.0 (San Diego, USA).

## Results

### Study subjects

Characteristics of all included control subjects (n = 9) and COPD patients (n = 7) are shown in Table 1. Predicted FEV_1_ was 102.2% (84-116) in control subjects and 19.3% (14-24) in COPD patients. All the COPD patients were classified as spirometric GOLD stage IV according to the GOLD guidelines. Eight controls were non-smokers, one control was an ex-smoker and all the COPD patients were ex-smokers. Primary distal fibroblast cultures were obtained from all control subjects and patients. As we tried to get as much information as possible from this limited set of well-characterized lung fibroblasts, the fibroblast numbers used from the different control and patient groups are written for each individual experiment.

### Presence of the IP receptor in lung tissue and lung fibroblasts

**Figure 1:**
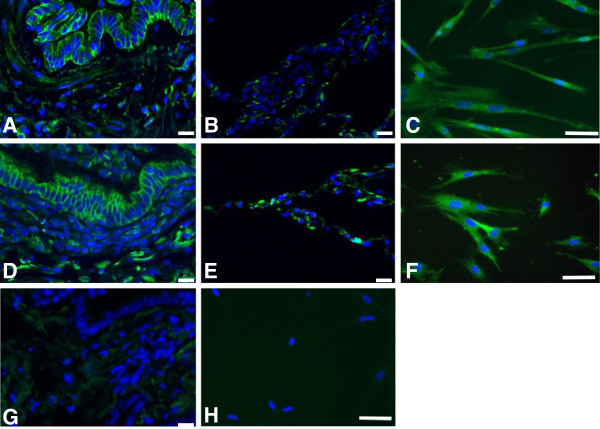
**The IP receptor is expressed by epithelial cells and fibroblasts in the lung.** Representative staining is shown in bronchial lung tissue (**A**), parencyhmal lung tissue (**B**), and distal lung fibroblasts *in vitro* (**C**) from control subjects and bronchial lung tissue (**D**), parenchymal lung tissue (**E**) and distal lung fibroblasts *in vitro* (**F**) from patients with COPD. Panel (**G**) and (**H**) show isotype-matched control IgG. Scale bars are indicated as 10 μm in Figure 1**A**, **B**, **D**, **E** and **G** and 50 μm in panel 1 **C**, **F** and **H**.

Immune positivity for the IP receptor was observed in epithelial, subepithelial and parenchymal cells in lung explants from control subjects (Figure 1A, B) and patients with COPD (Figure 1D, E). Both distal lung fibroblasts from control subjects (Figure 1C) and patients with COPD (Figure 1F) expressed the IP receptor. To confirm the specificity of primary antibody binding, isotype controls were used (Figure 1G and H).

### Prostacyclin and TGF-β_1_ production from distal lung fibroblasts

**Figure 2:**
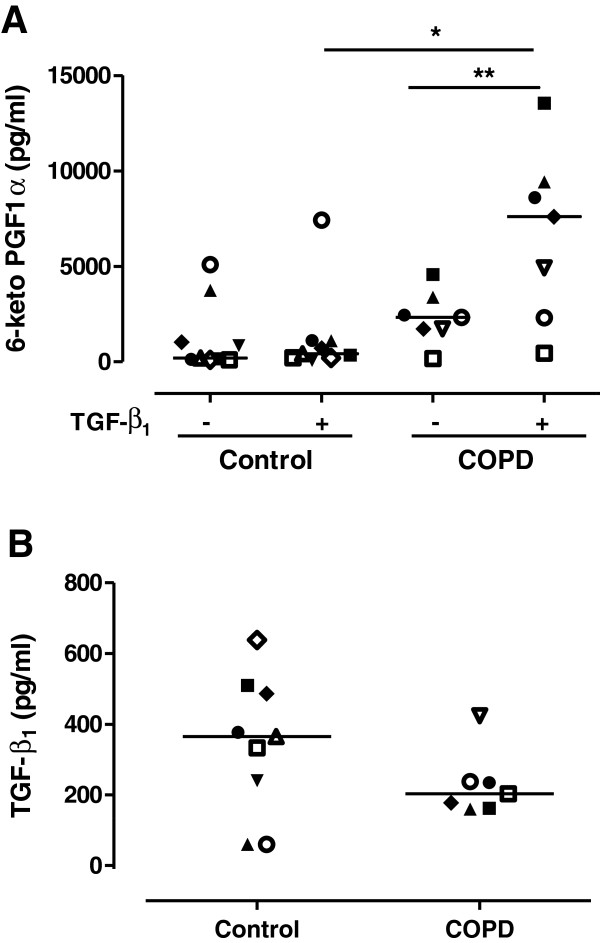
**A. Prostacyclin production is increased in COPD fibroblasts after TGF-β**_**1 **_**stimulation.** Prostacyclin synthesis was measured as 6-keto PGF_1α_ in the supernatant from distal lung fibroblasts from control subjects (n = 9) and patients with COPD (n = 7). Cells were stimulated with TGF-β_1_ (10 ng/ml) in 0.4% fibroblast medium. Data are presented as median with individual values and median values are represented as a line. *p < 0.05, **p < 0.01. Statistical analysis is performed with Mann Whitney test to compare differences between control and COPD fibroblasts and Wilcoxon paired rank sum test is used to analyze differences within the control and COPD fibroblasts after TGF-β_1_ stimulation. **B.** There is no difference in endogenous TGF-β_1_ production in control or COPD fibroblasts. TGF-β_1_ production measured in supernatant from distal lung fibroblasts from control subjects (n = 9) and patients with COPD (n = 7). Data are presented as median with individual values and median values are represented as a line. Statistical analysis is performed with Mann Whitney test to compare differences between control and COPD fibroblasts.

The stable prostacyclin metabolite 6-keto PGF_1α_ was 2.9-fold increased in COPD fibroblasts after stimulation with TGF-β_1_ (10 ng/ml) (n = 7, p = 0.007, Figure 2A). TGF-β_1_ did not alter prostacyclin production in fibroblasts from healthy controls (n = 9) and prostacyclin synthesis after TGF-β_1_ stimulation was significantly higher (p < 0.05) in lung fibroblasts from patients with COPD compared to control subjects (Figure 2A). There was no significant difference in the production of endogenously derived TGF-β_1_ in fibroblast medium from control subjects (n = 9) or COPD patients (n = 7) (Figure 2B).

### Iloprost reduces collagen I synthesis in distal lung fibroblasts

**Figure 3:**
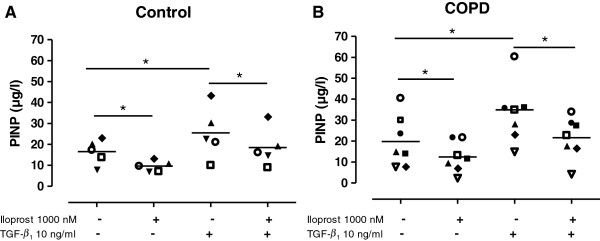
**Collagen synthesis is enhanced by TGF-β**_**1 **_**in control and COPD fibroblasts and attenuated by iloprost treatment.** Collagen I production was measured as the propeptide of type I procollagen (PINP) in the supernatant from distal lung fibroblasts from control subjects (n = 5; **A**) and patients with COPD (n = 7; **B**). Cells were stimulated with iloprost (1000 nM) and TGF-β_1_ (10 ng/ml) in 0.4% fibroblast medium pretreated with indomethacin (3 μM). Data are presented as median with individual values and median values are represented as a line. *p < 0.05. Statistical analysis is performed with Mann Whitney t-test to compare differences between control and COPD fibroblasts and Wilcoxon paired rank sum test is used to analyze differences within the control and COPD fibroblasts.

Collagen I synthesis was significantly reduced by iloprost (1000 nM) in lung fibroblasts obtained from control subjects (n = 5, p < 0.05, Figure 3A) and patients with COPD (n = 7, p < 0.05, Figure 3B). Addition of TGF-β_1_ enhanced collagen I synthesis in fibroblasts from both control subjects (p < 0.05) and COPD patients (p < 0.05). Subsequently, treatment with iloprost decreased collagen I production after TGF-β_1_ stimulation in fibroblasts from both controls (p < 0.05, Figure 3A) and COPD patients (p < 0.05, Figure 3B). However, there were no significant differences in collagen I synthesis before or after stimulation with TGF-β_1_ between fibroblasts from control subjects and COPD patients. Pretreatment with the COX inhibitor indomethacin did not affect the collagen I synthesis (data not shown).

### Iloprost alters proteoglycan production in fibroblasts from control subjects

**Figure 4:**
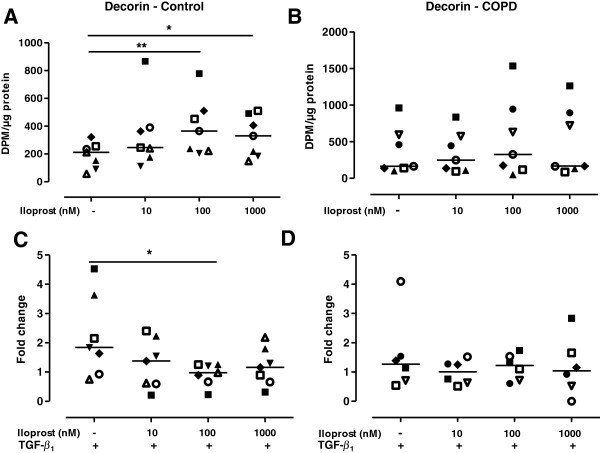
**Decorin production is altered by iloprost in fibroblasts from control subjects but not from COPD patients.** Decorin production in distal lung fibroblasts from control subjects (n = 7; **A**) and patients with COPD (n = 7; **B**). Cells were stimulated with iloprost (10, 100 or 1000 nM) and in combination with TGF-β_1_ (10 ng/ml) in 0.4% fibroblast medium pretreated with indomethacin (3 μM). Changes in decorin production after TGF-β_1_ stimulation are expressed as fold change compared to non-stimulated fibroblasts in 0.4% medium from control subjects (**C**) and COPD patients (**D**). Data are presented as median with individual values and median values are represented as a line. *p < 0.05; **p < 0.01. Statistical analysis is performed with ANOVA on ranks followed by a post hoc test to compare differences between control and COPD fibroblasts and within the control and COPD fibroblasts after iloprost and TGF-β_1_ stimulation.

**Figure 5:**
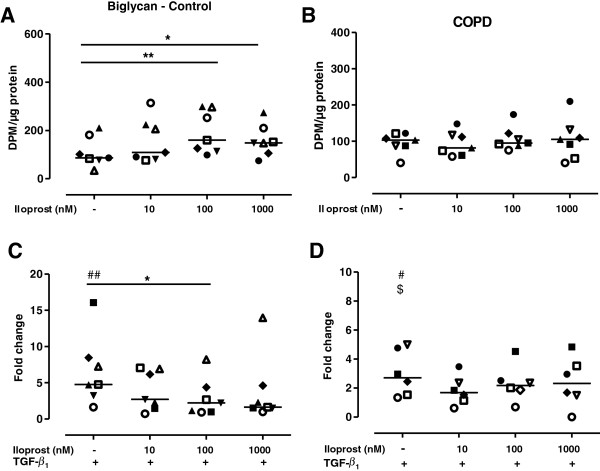
**Biglycan production is altered by iloprost in fibroblasts from control subjects but not from COPD patients.** Biglycan production in distal lung fibroblasts from control subjects (n = 7; **A**) and patients with COPD (n = 7; **B**). Cells were stimulated with iloprost (10, 100 or 1000 nM) and in combination with TGF-β_1_ (10 ng/ml) in 0.4% fibroblast medium pretreated with indomethacin (3 μM). Changes in biglycan production after TGF-β_1_ stimulation are expressed as fold change compared to non-stimulated fibroblasts in 0.4% medium from control subjects (**C**) and COPD patients (**D**). Data are presented as median with individual values and median values are represented as a line. *p < 0.05; **p < 0.01. ^#^p < 0.05 and ^##^p < 0.01 indicate significant differences in biglycan production after TGF-β_1_ stimulation compared to basal levels. ^$^p < 0.05 indicate significant difference in biglycan synthesis after TGF-β_1_ stimulation between control and COPD fibroblasts. Statistical analysis is performed with ANOVA on ranks followed by a post hoc test to compare differences between control and COPD fibroblasts and within the control and COPD fibroblasts after iloprost and TGF-β_1_ stimulation.

Further studies were performed to evaluate if iloprost also affected the collagen-associated proteoglycans biglycan and decorin. There were no differences in either decorin or biglycan synthesis between fibroblasts from control subjects (n = 7) and patients with COPD (n = 7). Addition of TGF-β_1_ (10 ng/ml) did not change the decorin production (Figure 4C and 4D), whereas TGF-β_1_ enhanced the production of biglycan 6.6-fold in fibroblasts from control subjects (p < 0.01; Figure 5C) and 3.0-fold in fibroblasts from COPD patients (p < 0.05; Figure 5D) and the biglycan production after TGF-β_1_ stimulation was significantly higher in fibroblasts from control subjects compared to fibroblast from COPD patients (p < 0.05). Pretreatment with indomethacin did not affect proteoglycan production in lung fibroblasts from either control subjects or COPD patients (data not shown). Iloprost (100 nM and 1000 nM) significantly enhanced the synthesis of decorin (Figure 4A) and biglycan (Figure 5A) in fibroblasts from control subjects. Furthermore, after TGF-β_1_ treatment, iloprost significantly attenuated the synthesis of both decorin (Figure 4C) and biglycan (Figure 5C) in fibroblasts from control subjects. However, in fibroblasts from COPD patients there was no significant effect of iloprost treatment on either decorin (Figure 4B and D) or biglycan (Figure 5B and 5D) production before or after TGF-β_1_ stimulation.

### Effect of iloprost on migratory capacity and proliferation rate

**Figure 6:**
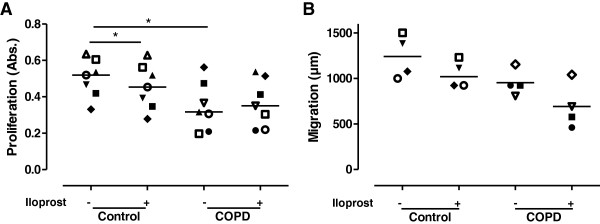
**Iloprost treatment did not change migration capacity or proliferative rate in control or COPD fibroblasts.** Proliferative rate of distal lung fibroblasts from control subjects (n = 7) and patients with COPD (n = 7) (**A**). All proliferation experiments are the mean of quintuplicate. Cells were stimulated with iloprost (100 nM) in fibroblast medium containing 0.4% serum pretreated with indomethacin (3 μM). Fibroblast medium containing 10% serum was used as internal control. Migration capacity in distal lung fibroblasts from control subjects (n = 4) and patients with COPD (n = 4), *p < 0.05 (**B**). All migration assays are the mean of triplicates. Data are presented as median with individual values and median values are represented as a line. Statistical analysis is performed with Mann Whitney test to compare differences between control and COPD fibroblasts and Wilcoxon paired rank sum test is used to analyze differences within the control and COPD fibroblasts after iloprost stimulation.

Iloprost (100 nM) significantly decreased the proliferative rate in fibroblasts from control subjects (n = 7, p < 0.05), whereas iloprost had no effect on proliferation rate in fibroblasts from COPD patients (n = 7) (Figure 6A). However, fibroblasts from COPD patients showed a reduced proliferative rate compared to control fibroblasts (p < 0.05). Although, there was a tendency to reduced migration capacity after iloprost treatment, iloprost (100 nM) did not significantly affect the migratory capacity in either fibroblasts from COPD patients (n = 4) or control subjects (n = 4) (Figure 6B). Fibroblasts from COPD patients showed a reduced migratory capacity (866 ± 145 *vs* 1309 ± 31; p < 0.05) compared to fibroblasts from COPD patients.

### Effect of iloprost on contractile capacity

**Figure 7:**
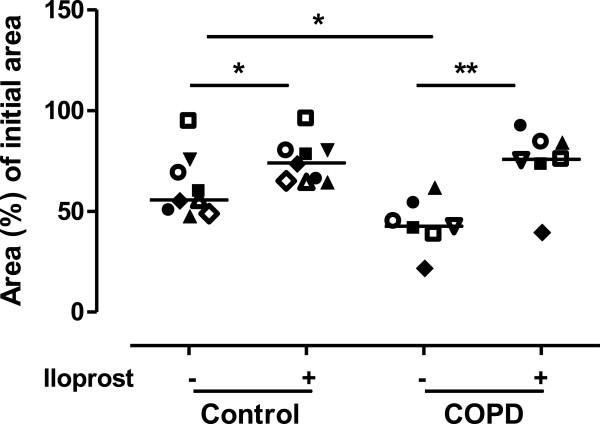
**Iloprost reduced fibroblast gel contractions in control and COPD fibroblasts.** Fibroblast gel contractions were performed with lung fibroblasts from control subjects (n = 8) and patients with COPD (n = 7). Cells were stimulated with iloprost (1000 nM) in fibroblast medium containing 0.4% serum and indomethacin (3 μM). All gel contraction experiments are the mean of triplicates and measured after 48 h. Data are presented as median with individual values and median values are represented as a line. *p < 0.05; **p < 0.01. Statistical analysis is performed with Mann Whitney test to compare differences between control and COPD fibroblasts and Wilcoxon paired rank sum test is used to analyze differences within the control and COPD fibroblasts after iloprost stimulation.

Iloprost (1000 nM) attenuated the contractile capacity in fibroblasts from both COPD patients (n = 7, p < 0.01) and control subjects (n = 9, p < 0.05). Importantly, fibroblasts from COPD patients showed a more contractile phenotype then fibroblasts from control subjects (p < 0.05) (Figure 7).

## Discussion

In the present study we show that distal lung fibroblasts respond to prostacyclin, and that prostacyclin may alter fibroblast activity and thereby remodeling processes. Interestingly, lung fibroblasts from patients with COPD had a higher synthesis of prostacyclin compared to control fibroblasts. The prostacyclin analog iloprost decreased collagen I synthesis and contractile capacity in both fibroblasts from control subjects and COPD, whereas alterations in proteoglycan production and proliferative rate were only present in fibroblasts from control subjects. The present data implicate an important issue that severe COPD patients may have a reduced repair mechanism in the ECM structure of the collagen network in the distal lung. Previous studies support our findings that changes in ECM synthesis are involved in pathologic conditions of COPD [17] and that fibroblasts from COPD patients may have a reduced or defective capacity of tissue repair [17,18,28]. Conversely, in this study, fibroblasts from COPD patients showed not only an altered production of prostacyclin and proteoglycans, but also a general decrease in proliferative rate and migratory capacity and an increased contractile phenotype compared to fibroblasts from control subjects. The capacity of fibroblasts to respond to an injury through the production or inhibition of mediators as TGF-β_1_ and prostacyclin may determine the nature of the repair responses involving ECM homeostasis. Notably, TGF-β_1_ is involved in remodeling processes in COPD through an activation of fibroblasts and induction of ECM production and may regulate proteoglycan synthesis [17,29]. TGF-β_1_ expression has previously been shown to be increased in central airways [30] and in peripheral blood [31] from COPD patients. Also, fibroblasts from peribronchiolar areas of lung tissue from patients with severe emphysema have increased production of TGF-β_1_[32]. However, in our study, we could not detect any significant differences in TGF-β_1_ synthesis between distal fibroblasts obtained from COPD patients and control subjects, indicating that the synthesis of TGF-β_1_ may be dependent on cellular origin and also location in the lung. In the present study, we could show that TGF-β_1_ increased the production of the ECM proteins collagen I and biglycan in distal lung fibroblasts derived from both controls and COPD patients. However, there was no difference in collagen I synthesis between fibroblasts from control and COPD patients. Corresponding with our data, Krimmer et al did not detect any differences in fibrillar collagen between non-COPD and COPD fibroblasts after TGF-β_1_ stimulation; neither did cigarette smoke extract affect the fibroblast ability to deposit collagen [33]. Noordhoek et al did also not find any differences in collagen I synthesis between parenchymal fibroblasts from patients with mild emphysema and patients with severe emphysema [34], implicating that collagen I synthesis probably is preserved in fibroblasts from COPD patients. Interestingly, in the present study, prostacyclin synthesis was significantly increased after TGF-β_1_ stimulation in distal lung fibroblast from COPD patients. In line with these findings, TGF-β_1_ stimulation increased both COX expression and enhanced prostacyclin synthesis in a human lung fibroblast cell line [35]. In contrast, decreased levels of prostacyclin production have been found in distal lung fibroblasts from patients with interstitial pulmonary fibrosis [15]. These data imply that alterations in the levels of prostacyclin may be a marker of ongoing remodeling processes. Prostacyclin has previously been associated with tissue repair and remodeling processes by inhibiting profibrotic responses of fibroblasts [9,10,36] and attenuating pulmonary fibrosis in animal models [16,37]. In the present study, the prostacyclin analog iloprost reduced collagen synthesis and subsequently attenuated the increased collagen production in response to TGF-β_1_. These results correspond to other findings where prostacyclin down regulated collagen synthesis in rat cardiac fibroblasts [38]. Nonetheless, we could not detect any significant differences in collagen I production between fibroblasts from COPD patients or control subjects after iloprost treatment. Conversely, in the present study, iloprost balanced the changes in collagen I synthesis by altering the production of the collagen-associated proteoglycans decorin and biglycan in fibroblasts from control subjects, but iloprost had no effect on these proteoglycans in fibroblasts from COPD patients. Decorin is thought to be a negative regulator of TGF-β_1_ by binding and neutralizing significant amounts of this growth factor [39]. Decorin and biglycan also shape and complement the collagen fibril structure, and decorin mediates the binding of collagen fibers [40]. Our data indicate that fibroblasts from COPD patients may have a defective repair mechanism in the collagen network fibrillogenesis. Thus, high levels of prostacyclin could generate reduced collagen synthesis that is not regulated and stabilized by decorin or biglycan, which thereby may accelerate the formation of emphysematous tissue in COPD. In line with the findings of Hallgren et al [17], we found that biglycan synthesis was reduced by the distally-derived fibroblasts from severe COPD patients, whereas there were no differences in decorin synthesis before or after TGF-β_1_ stimulation between COPD and control fibroblasts. However, decorin synthesis has been shown to be decreased in distal fibroblasts from patients with severe emphysema [34] and decorin gene expression has also been shown to be decreased in centrally-derived lung fibroblasts from patients with severe COPD [41]. Decreased decorin and biglycan expressions have also been shown in peribronchiolar areas in patients with severe pulmonary emphysema [17,32]. One explanation to these findings could be that decorin is regulated differently at mRNA levels and protein levels with a higher turnover rate at protein levels [21]. Mice that lack decorin have dysfunctional collagen fibrils with reduced tensile strength [42] and show altered lung mechanical properties, as enhanced lung compliance [43]. Subsequently, decorin has been shown to reduce lung fibrosis induced by TGF-β_1_[44]. Hypothetically, taken all these data together, fibroblasts from COPD patients may have an imbalance in the regulatory properties of the collagen network homeostasis, indicating that proteoglycan production is dysregulated in the collagen network assembly in response to iloprost or TGF-β_1_. Matrix metalloproteinases (MMPs) are essential for the degradation of ECM, and MMP-9 has been shown to be upregulated in severe COPD [45]. Notably, MMP-9 release appears to be resistant to glucocorticoid therapy [45]. Interestingly, prostacyclin treatment attenuated MMP-9 synthesis in mesangial cells [46] and in an animal model of cigarette smoke induced emphysema [47]. Unfortunately, we could not detect any MMP-9 synthesis in the present study. Importantly, distally-derived fibroblasts from severe COPD patients demonstrated a more contractile phenotype in this study, probably due to enhanced ROCK1 activity [18], than fibroblasts from control subjects, and treatment with iloprost attenuated the contractile capacity to the same level as fibroblasts from control subjects in the present study. The inhibitory effect of prostacyclin analogs on fibroblast gel contractions has previously been shown in healthy lung fibroblasts and the response was mediated through cAMP activation of PKA [48]. Proliferative rate was decreased by iloprost in control fibroblast, whereas COPD patients had a generally reduced proliferative rate that was not affected by iloprost treatment. Fibroblasts from COPD patients have also previously been reported to have reduced proliferative capacity [17,49], which may contribute to the emphysema formation in the distal COPD lung. In the present study, control fibroblasts were obtained from healthy subjects of mixed age. Changes in ECM components in this study may reflect ongoing natural aging processes [50] and it has been shown that aging processes in the lung may occur independently of emphysema formation related to COPD pathogenesis [51]. We could not detect any differences in studied parameters due to age within the control group; neither could we detect any differences in studied parameters due to different sampling techniques, transbronchial biopsies versus lung explants, implicating that the alterations presented in this study is probably linked to disease, and not to aging. Despite the limited numbers of observations in the present study, we could support the findings in a parallel study published by Hallgren et al [17] that the different sampling techniques and the age distribution in the two study populations did not interfere with the obtained results. Medical treatments may also influence the fibroblasts obtained from the COPD patients. It would have been an advantage if we had lung function data from all the controls. On the other hand, the donor lungs from the healthy individuals had been judged by the clinicians to be suitable for lung transplantation and we received the lungs due to the fact that they could not find any matching recipients at the moment. It is well known that smoking and ex-smoking may have an impact on fibroblast function. However, the fibroblasts obtained from the former smoker lung did not differ from the other fibroblast controls in the studied parameters, neither could Hallgren et al. find any differences [18]. Studies on smokers and patients in different GOLD stages will be performed in the future to further investigate the importance of prostacyclin in remodeling processes.

## Conclusions

Iloprost reduced collagen I synthesis and fibroblast contractility but did not affect the collagen-associated proteoglycans or proliferative capacity in fibroblasts from COPD patients. In addition, fibroblasts from COPD patients had a reduced proliferative rate and migration, decreased biglycan synthesis and an increased contractile capacity compared to fibroblasts from control subjects. Our data imply that COPD patients may have an altered fibroblast function and defect repair mechanism in the ECM structure of the collagen network assembly. Due to the altered fibroblast function, patients with COPD may not be able to maintain normal tissue repair capacity. The prostacyclin analog iloprost appears to promote an anti-fibrotic phenotype with reduced collagen synthesis in pulmonary fibroblasts that may enhance the severity of emphysema formation in COPD. These findings should be considered when administrating iloprost to patients with severe COPD.

## Abbreviations

COPD: Chronic obstructive pulmonary disease;COX: Cyclooxygenase;IP receptor: Prostacyclin receptor;MMP: Matrix metalloproteinase;PINP: Propeptide of type 1 procollagen;PG: Prostaglandin;TGF-β1: Transforming growth factor-β_1_

## Competing interests

The authors state no competing interests.

## Authors’ contributions

AKL conceived and designed the study, carried out the cell culture stimulations, analysis of prostacyclin and TGF-β_1_ production, analysis of different proteoglycan production and proliferation rate on primary lung fibroblasts, performed the statistical analysis, interpretation of data and drafted the manuscript. OH isolated fibroblasts, carried out the collagen gel contraction assays, participated in the interpretation of data and helped to draft the manuscript. AAS performed the immunohistochemistry and helped to draft the manuscript. LT carried out some of the cell culture works and performed the SDS-PAGE for analysis of proteoglycans. JB and JK carried out the migration studies. KN isolated fibroblasts and participated in the interpretation of data. LB participated in the interpretation of data and helped to draft the manuscript. CGL participated in the design of the study, interpretation of data and helped to draft the manuscript. GWT participated in the design of the study, interpretation of data and helped to draft the manuscript. All authors read and approved the final manuscript.

## References

[B1] Postma DS Timens W Remodeling in asthma and chronic obstructive pulmonary disease Proc Am Thorac Soc 2006 3 434 439 10.1513/pats.200601-006AW 16799088

[B2] Siafakas NM Antoniou KM Tzortzaki EG Role of angiogenesis and vascular remodeling in chronic obstructive pulmonary disease Int J Chron Obstruct Pulmon Dis 2007 2 453 462 18268919 PMC2699970

[B3] Barbera JA Blanco I Pulmonary hypertension in patients with chronic obstructive pulmonary disease: advances in pathophysiology and management Drugs 2009 69 1153 1171 10.2165/00003495-200969090-00002 19537834

[B4] Gessler T Seeger W Schmehl T Inhaled prostanoids in the therapy of pulmonary hypertension J Aerosol Med Pulm Drug Deliv 2008 21 1 12 10.1089/jamp.2007.0657 18518827

[B5] Dernaika TA Beavin M Kinasewitz GT Iloprost improves gas exchange and exercise tolerance in patients with pulmonary hypertension and chronic obstructive pulmonary disease Respiration 2010 79 377 382 10.1159/000242498 19786728

[B6] Hegewald MJ Elliott CG Sustained improvement with iloprost in a COPD patient with severe pulmonary hypertension Chest 2009 135 536 537 10.1378/chest.08-1515 19201716

[B7] Moncada S Higgs EA Vane JR Human arterial and venous tissues generate prostacyclin (prostaglandin x), a potent inhibitor of platelet aggregation Lancet 1977 1 18 20 63657 10.1016/s0140-6736(77)91655-5

[B8] Coleman RA Smith WL Narumiya S International Union of Pharmacology classification of prostanoid receptors: properties, distribution, and structure of the receptors and their subtypes Pharmacol Rev 1994 46 205 229 7938166

[B9] Stratton R Shiwen X Martini G Holmes A Leask A Haberberger T Martin GR Black CM Abraham D Iloprost suppresses connective tissue growth factor production in fibroblasts and in the skin of scleroderma patients J Clin Invest 2001 108 241 250 11457877 10.1172/JCI12020 PMC203022

[B10] Kohyama T Liu X Kim HJ Kobayashi T Ertl RF Wen FQ Takizawa H Rennard SI Prostacyclin analogs inhibit fibroblast migration Am J Physiol Lung Cell Mol Physiol 2002 283 L428 432 12114205 10.1152/ajplung.00432.2001

[B11] Hogg JC Pathophysiology of airflow limitation in chronic obstructive pulmonary disease Lancet 2004 364 709 721 10.1016/S0140-6736(04)16900-6 15325838

[B12] Westergren-Thorsson G Larsen K Nihlberg K Andersson-Sjoland A Hallgren O Marko-Varga G Bjermer L Pathological airway remodelling in inflammation Clin Respir J 2010 4 Suppl 1 1 8 20500603 10.1111/j.1752-699X.2010.00190.x

[B13] Fine A Poliks CF Smith BD Goldstein RH The accumulation of type I collagen mRNAs in human embryonic lung fibroblasts stimulated by transforming growth factor-beta Connect Tissue Res 1990 24 237 247 10.3109/03008209009152152 2376126

[B14] Polgar P Taylor L Alterations in prostaglandin synthesis during senescence of human lung fibroblasts Mech Ageing Dev 1980 12 305 310 10.1016/0047-6374(80)90063-9 6993800

[B15] Cruz-Gervis R Stecenko AA Dworski R Lane KB Loyd JE Pierson R King G Brigham KL Altered prostanoid production by fibroblasts cultured from the lungs of human subjects with idiopathic pulmonary fibrosis Respir Res 2002 3 17 10.1186/rr166 11980586 PMC107846

[B16] Lovgren AK Jania LA Hartney JM Parsons KK Audoly LP Fitzgerald GA Tilley SL Koller BH COX-2-derived prostacyclin protects against bleomycin-induced pulmonary fibrosis Am J Physiol Lung Cell Mol Physiol 2006 291 L144 156 10.1152/ajplung.00492.2005 16473862

[B17] Hallgren O Nihlberg K Dahlback M Bjermer L Eriksson LT Erjefalt JS Lofdahl CG Westergren-Thorsson G Altered fibroblast proteoglycan production in COPD Respir Res 2010 11 55 10.1186/1465-9921-11-55 20459817 PMC2886021

[B18] Hallgren O Rolandsson S Andersson-Sjoland A Nihlberg K Wieslander E Kvist-Reimer M Dahlback M Eriksson L Bjermer L Erjefalt J Enhanced ROCK1 dependent contractility in fibroblast from chronic obstructive pulmonary disease patients J Transl Med 2012 10 171 10.1186/1479-5876-10-171 22913419 PMC3477051

[B19] Nihlberg K Andersson-Sjoland A Tufvesson E Erjefalt JS Bjermer L Westergren-Thorsson G Altered matrix production in the distal airways of individuals with asthma Thorax 2010 65 670 676 10.1136/thx.2009.129320 20685740

[B20] Tufvesson E Westergren-Thorsson G Alteration of proteoglycan synthesis in human lung fibroblasts induced by interleukin-1beta and tumor necrosis factor-alpha J Cell Biochem 2000 77 298 309 10.1002/(SICI)1097-4644(20000501)77:2<298::AID-JCB12>3.0.CO;2-D 10723095

[B21] Tiedemann K Malmstrom A Westergren-Thorsson G Cytokine regulation of proteoglycan production in fibroblasts: separate and synergistic effects Matrix Biol 1997 15 469 478 10.1016/S0945-053X(97)90020-2 9106158

[B22] Tufvesson E Malmstrom J Marko-Varga G Westergren-Thorsson G Biglycan isoforms with differences in polysaccharide substitution and core protein in human lung fibroblasts Eur J Biochem 2002 269 3688 3696 10.1046/j.1432-1033.2002.03058.x 12153565

[B23] Westergren-Thorsson G Sarnstrand B Fransson LA Malmstrom A TGF-beta enhances the production of hyaluronan in human lung but not in skin fibroblasts Exp Cell Res 1990 186 192 195 10.1016/0014-4827(90)90227-2 2298235

[B24] Gillies RJ Didier N Denton M Determination of cell number in monolayer cultures Anal Biochem 1986 159 109 113 10.1016/0003-2697(86)90314-3 3812988

[B25] Westergren-Thorsson G Persson S Isaksson A Onnervik PO Malmstrom A Fransson LA L-iduronate-rich glycosaminoglycans inhibit growth of normal fibroblasts independently of serum or added growth factors Exp Cell Res 1993 206 93 99 10.1006/excr.1993.1124 7683278

[B26] Larsen K Tufvesson E Malmstrom J Morgelin M Wildt M Andersson A Lindstrom A Malmstrom A Lofdahl CG Marko-Varga G Presence of activated mobile fibroblasts in bronchoalveolar lavage from patients with mild asthma Am J Respir Crit Care Med 2004 170 1049 1056 10.1164/rccm.200404-507OC 15256392

[B27] Gullberg D Tingstrom A Thuresson AC Olsson L Terracio L Borg TK Rubin K Beta 1 integrin-mediated collagen gel contraction is stimulated by PDGF Exp Cell Res 1990 186 264 272 10.1016/0014-4827(90)90305-T 2298242

[B28] Togo S Holz O Liu X Sugiura H Kamio K Wang X Kawasaki S Ahn Y Fredriksson K Skold CM Lung fibroblast repair functions in patients with chronic obstructive pulmonary disease are altered by multiple mechanisms Am J Respir Crit Care Med 2008 178 248 260 10.1164/rccm.200706-929OC 18467512 PMC2542423

[B29] Coker RK Laurent GJ Pulmonary fibrosis: cytokines in the balance Eur Respir J 1998 11 1218 1221 10.1183/09031936.98.11061218 9657557

[B30] Kokturk N Tatlicioglu T Memis L Akyurek N Akyol G Expression of transforming growth factor beta1 in bronchial biopsies in asthma and COPD J Asthma 2003 40 887 893 10.1081/JAS-120023580 14736088

[B31] Stoll P Wuertemberger U Bratke K Zingler C Virchow CJ Lommatzsch M Stage-dependent association of BDNF and TGF-beta1 with lung function in stable COPD Respir Res 2012 13 116 10.1186/1465-9921-13-116 23245944 PMC3561140

[B32] van Straaten JF Coers W Noordhoek JA Huitema S Flipsen JT Kauffman HF Timens W Postma DS Proteoglycan changes in the extracellular matrix of lung tissue from patients with pulmonary emphysema Mod Pathol 1999 12 697 705 10430274

[B33] Krimmer DI Burgess JK Wooi TK Black JL Oliver BG Matrix proteins from smoke-exposed fibroblasts are pro-proliferative Am J Respir Cell Mol Biol 2012 46 34 39 10.1165/rcmb.2010-0426OC 21778414

[B34] Noordhoek JA Postma DS Chong LL Menkema L Kauffman HF Timens W van Straaten JF van der Geld YM Different modulation of decorin production by lung fibroblasts from patients with mild and severe emphysema COPD 2005 2 17 25 10.1081/COPD-200050678 17136957

[B35] Jackson BA Goldstein RH Roy R Cozzani M Taylor L Polgar P Effects of transforming growth factor beta and interleukin-1 beta on expression of cyclooxygenase 1 and 2 and phospholipase A2 mRNA in lung fibroblasts and endothelial cells in culture Biochem Biophys Res Commun 1993 197 1465 1474 10.1006/bbrc.1993.2642 8280164

[B36] Van Ly D Burgess JK Brock TG Lee TH Black JL Oliver BG Prostaglandins but not leukotrienes alter extracellular matrix protein deposition and cytokine release in primary human airway smooth muscle cells and fibroblasts Am J Physiol Lung Cell Mol Physiol 2012 303 L239 250 10.1152/ajplung.00097.2012 22637153

[B37] Zhu Y Liu Y Zhou W Xiang R Jiang L Huang K Xiao Y Guo Z Gao J A prostacyclin analogue, iloprost, protects from bleomycin-induced pulmonary fibrosis in mice Respir Res 2010 11 34 10.1186/1465-9921-11-34 20302663 PMC2848635

[B38] Liu X Thangavel M Sun SQ Kaminsky J Mahautmr P Stitham J Hwa J Ostrom RS Adenylyl cyclase type 6 overexpression selectively enhances beta-adrenergic and prostacyclin receptor-mediated inhibition of cardiac fibroblast function because of colocalization in lipid rafts Naunyn Schmiedebergs Arch Pharmacol 2008 377 359 369 10.1007/s00210-007-0196-0 17934720 PMC2665709

[B39] Yamaguchi Y Mann DM Ruoslahti E Negative regulation of transforming growth factor-beta by the proteoglycan decorin Nature 1990 346 281 284 10.1038/346281a0 2374594

[B40] Orgel JP Eid A Antipova O Bella J Scott JE Decorin core protein (decoron) shape complements collagen fibril surface structure and mediates its binding PLoS One 2009 4 e7028 10.1371/journal.pone.0007028 19753304 PMC2737631

[B41] Zandvoort A Postma DS Jonker MR Noordhoek JA Vos JT Timens W Smad gene expression in pulmonary fibroblasts: indications for defective ECM repair in COPD Respir Res 2008 9 83 10.1186/1465-9921-9-83 19087346 PMC2613883

[B42] Danielson KG Baribault H Holmes DF Graham H Kadler KE Iozzo RV Targeted disruption of decorin leads to abnormal collagen fibril morphology and skin fragility J Cell Biol 1997 136 729 743 10.1083/jcb.136.3.729 9024701 PMC2134287

[B43] Fust A LeBellego F Iozzo RV Roughley PJ Ludwig MS Alterations in lung mechanics in decorin-deficient mice Am J Physiol Lung Cell Mol Physiol 2005 288 L159 166 15447936 10.1152/ajplung.00089.2004

[B44] Kolb M Margetts PJ Sime PJ Gauldie J Proteoglycans decorin and biglycan differentially modulate TGF-beta-mediated fibrotic responses in the lung Am J Physiol Lung Cell Mol Physiol 2001 280 L1327 1334 11350814 10.1152/ajplung.2001.280.6.L1327

[B45] Vlahos R Wark PA Anderson GP Bozinovski S Glucocorticosteroids differentially regulate MMP-9 and neutrophil elastase in COPD PLoS One 2012 7 e33277 10.1371/journal.pone.0033277 22413009 PMC3296684

[B46] Kitahara M Ichikawa M Kinoshita T Shiozawa S Shigematsu H Komiyama A Prostacyclin inhibits the production of MMP-9 induced by phorbol ester through protein kinase A activation, but does not affect the production of MMP-2 in Human cultured mesangial cells Kidney Blood Press Res 2001 24 18 26 10.1159/000054201 11174002

[B47] Chen Y Hanaoka M Chen P Droma Y Voelkel NF Kubo K Protective effect of beraprost sodium, a stable prostacyclin analog, in the development of cigarette smoke extract-induced emphysema Am J Physiol Lung Cell Mol Physiol 2009 296 L648 656 10.1152/ajplung.90270.2008 19201816

[B48] Kamio K Liu X Sugiura H Togo S Kobayashi T Kawasaki S Wang X Mao L Ahn Y Hogaboam C Prostacyclin analogs inhibit fibroblast contraction of collagen gels through the cAMP-PKA pathway Am J Respir Cell Mol Biol 2007 37 113 120 10.1165/rcmb.2007-0009OC 17363776 PMC1899347

[B49] Noordhoek JA Postma DS Chong LL Vos JT Kauffman HF Timens W van Straaten JF Different proliferative capacity of lung fibroblasts obtained from control subjects and patients with emphysema Exp Lung Res 2003 29 291 302 10.1080/01902140303789 12746043

[B50] Janssens JP Pache JC Nicod LP Physiological changes in respiratory function associated with ageing Eur Respir J 1999 13 197 205 10.1183/09031936.99.14614549 10836348

[B51] Fukuchi Y The aging lung and chronic obstructive pulmonary disease: similarity and difference Proc Am Thorac Soc 2009 6 570 572 10.1513/pats.200909-099RM 19934351

